# Supplementary figures and images for: Divergent downstream biosynthetic pathways are supported by L-cysteine synthases of Mycobacterium tuberculosis
Source: eLife. 2024 Aug 29;12:RP91970. doi: 10.7554/eLife.91970 (PMC11361707; doi:10.7554/eLife.91970)

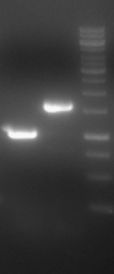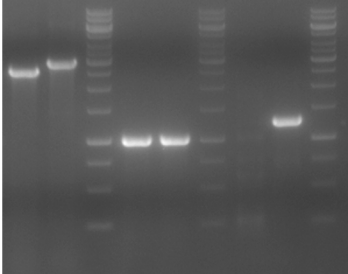

Supplement: Figure 1—source data 2. [file elife-91970-fig1-data2.pdf]

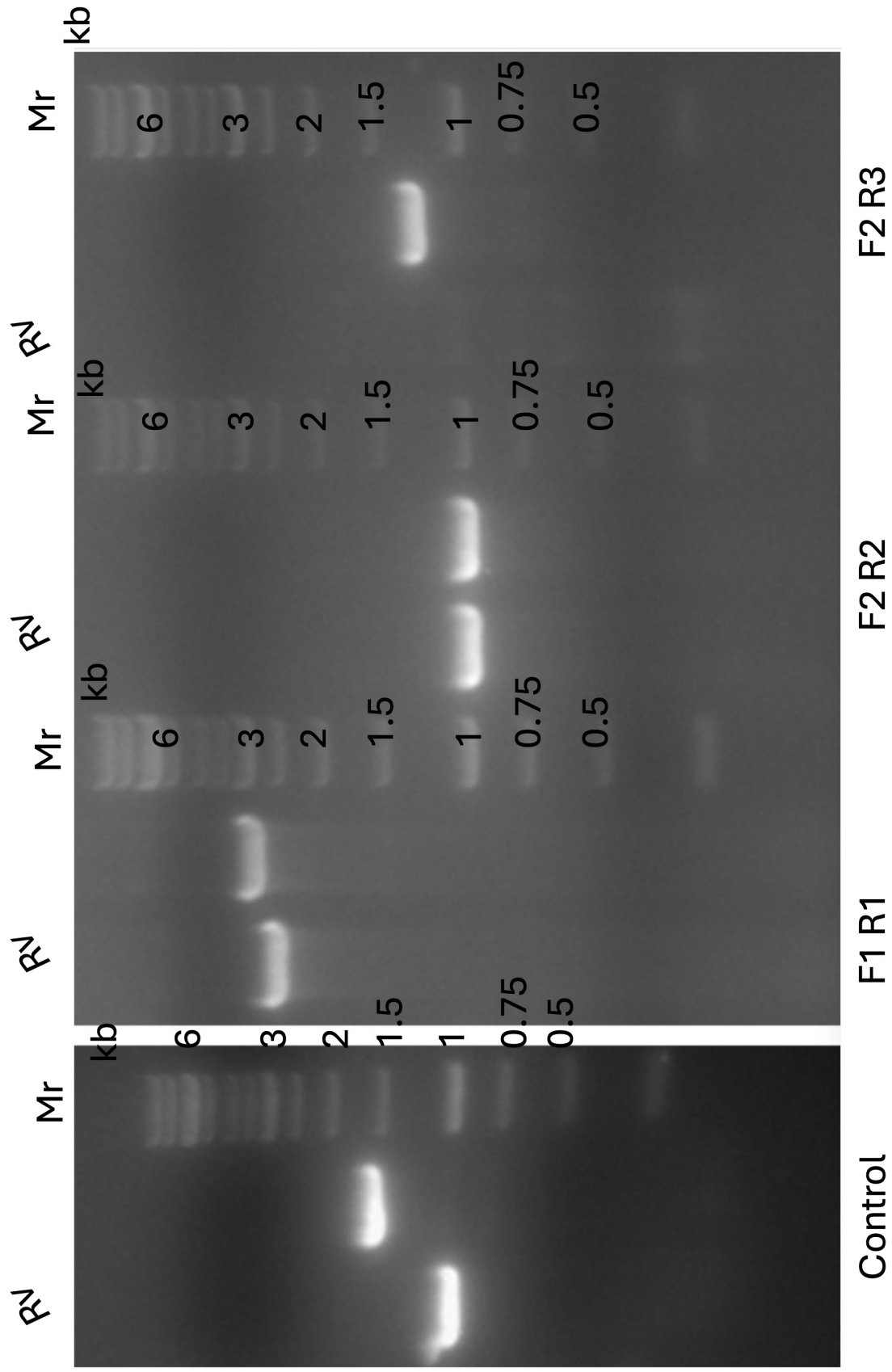

Supplement: Figure 1—source data 3. [file elife-91970-fig1-data3.pdf]

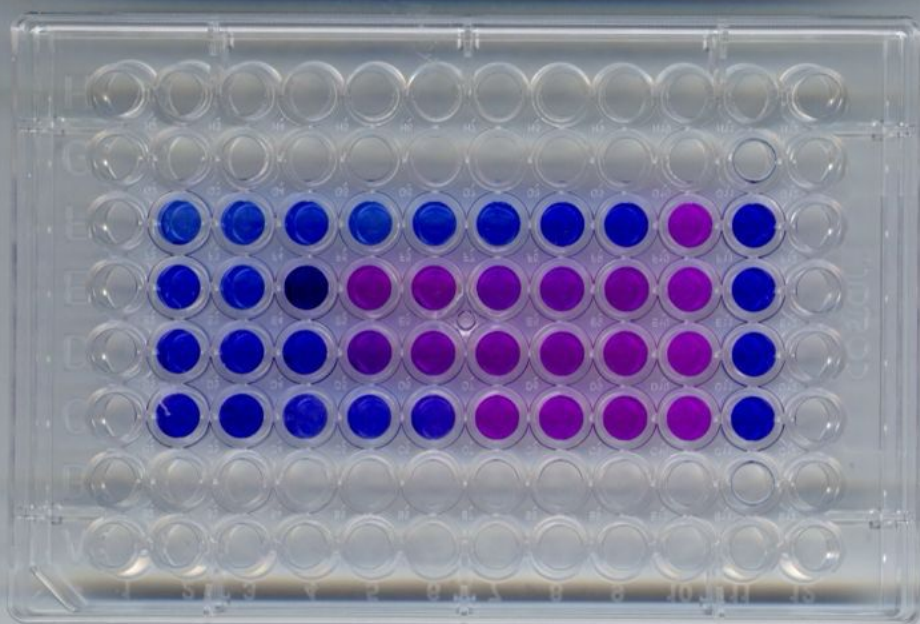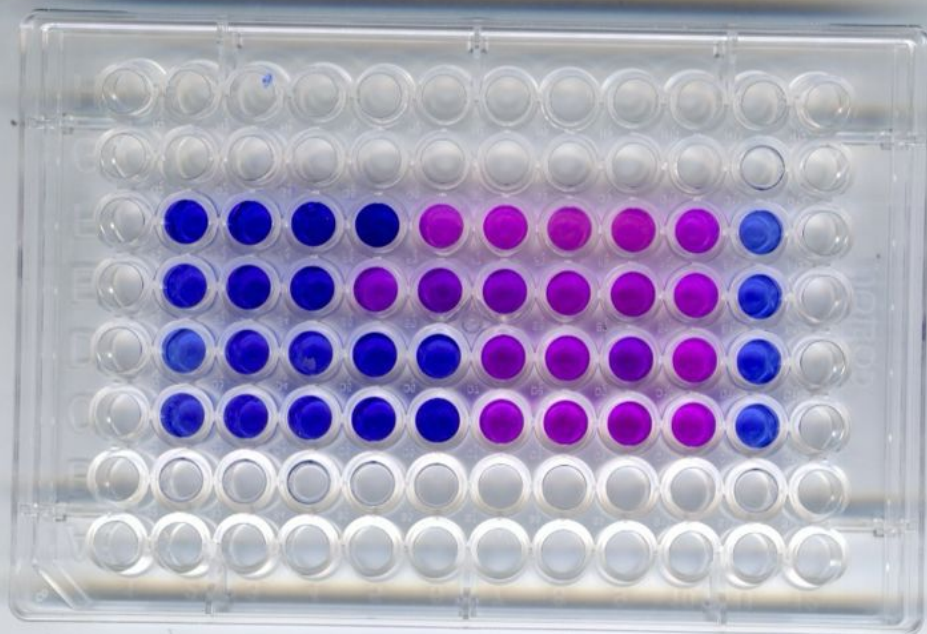

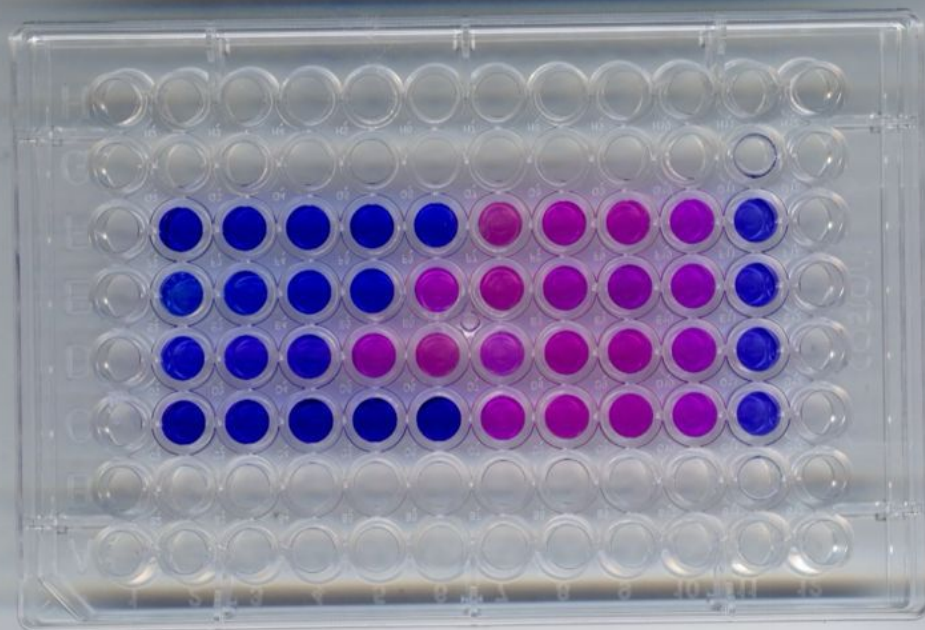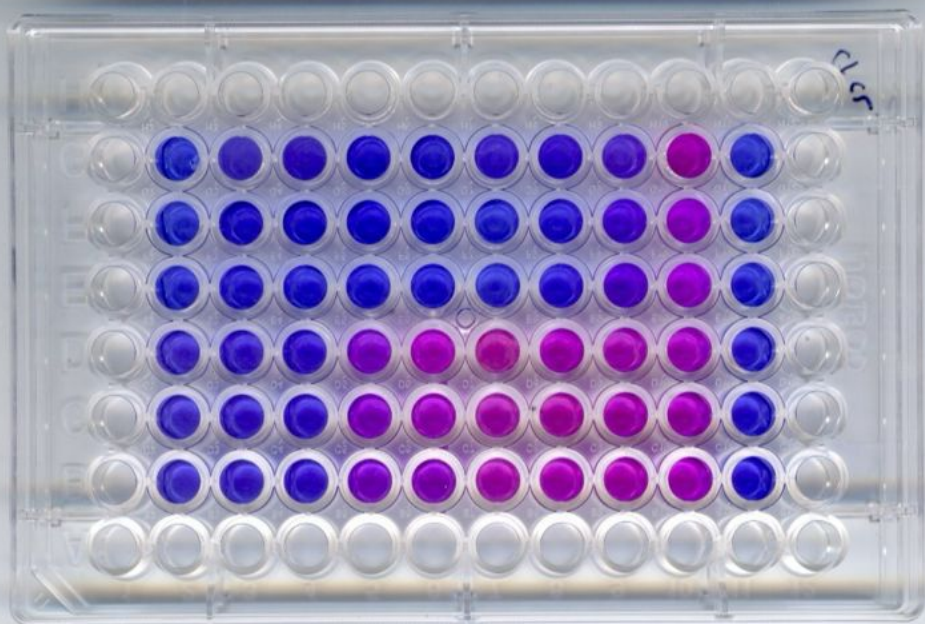

Supplement: Figure 6—source data 3. [file elife-91970-fig6-data3.pdf]
