# Supplementary material for: Divergent downstream biosynthetic pathways are supported by L-cysteine synthases of Mycobacterium tuberculosis
Source: eLife. 2024 Aug 29;12:RP91970. doi: 10.7554/eLife.91970 (PMC11361707; doi:10.7554/eLife.91970)
Supplement: Figure 6—source data 2. [file elife-91970-fig6-data2.pdf]

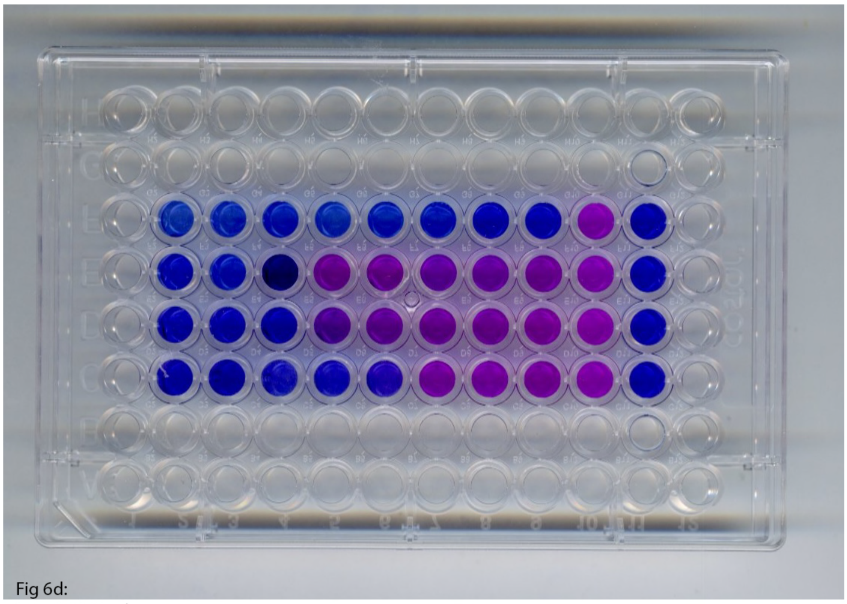

Fig 6d:
Row 1. INH only
Row 2. C1 Only
Row 3: MIC of C1+ serial 2-fold dilution of INH from MIC
Row 4: MIC of INH + serial 2-fold dilution of C1 from MIC

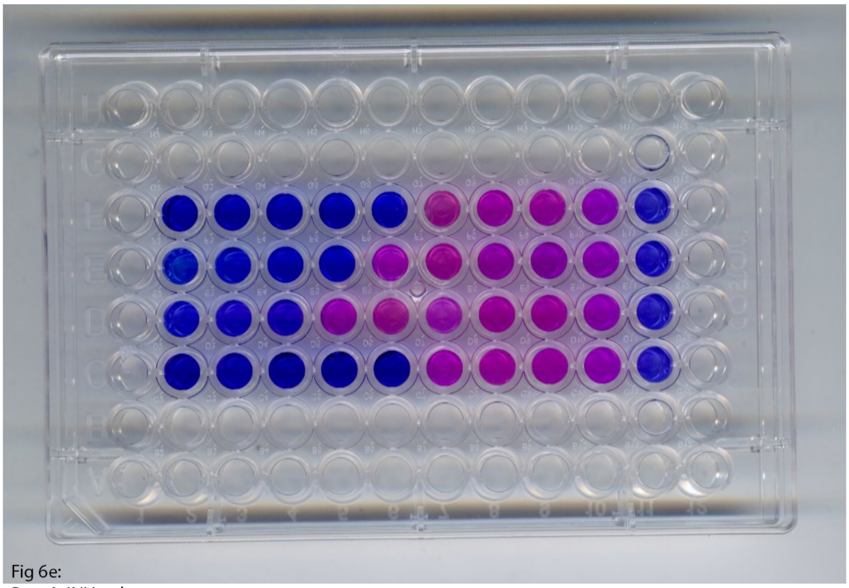

Fig 6e:
Row 1. INH only
Row 2. C2 Only
Row 3: MIC of C2+ serial 2-fold dilution of INH from MIC
Row 4: MIC of INH + serial 2-fold dilution of C2 from MIC

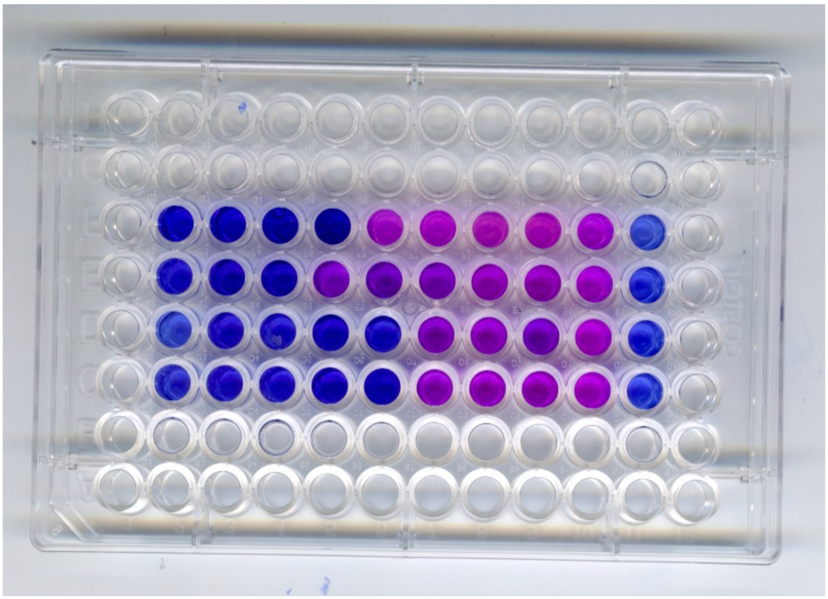

Fig 6f:
Row 1. INH only
Row 2. Compound C3
Row 3: MIC of C3 + serial 2-fold dilution of INH from MIC
Row 4: MIC of INH + serial 2-fold dilution of C3 from MIC
